# Whole Exome Sequencing Reveals a Novel APOE Mutation in a Patient With Sporadic Early-Onset Alzheimer's Disease

**DOI:** 10.3389/fneur.2022.899644

**Published:** 2022-06-10

**Authors:** Jaya Bagaria, Yeonsil Moon, Eva Bagyinszky, Kyu Hwan Shim, Seong Soo A. An, SangYun Kim, Seol Heui Han

**Affiliations:** ^1^Department of Bionanotechnology, Gachon University, Seongnam-si, South Korea; ^2^Department of Neurology, Konkuk University School of Medicine and Konkuk University Medical Center, Seoul, South Korea; ^3^Department of Industrial and Environmental Engineering, Graduate School of Environment, Gachon University, Seongnam, South Korea; ^4^Department of Neurology, Seoul National University College of Medicine and Seoul National University Budang Hospital, Seongnam-si, South Korea

**Keywords:** Low-Density Lipoprotein Receptor (LDLR), APOE, Leu159Pro, early-onset Alzheimer's disease, novel mutation, Whole Exome Sequence (WES) analysis

## Abstract

Apolipoprotein (APOE) is implicated and verified as the main risk factor for early-onset Alzheimer's disease (AD). APOE is a protein that binds to lipids and is involved in cholesterol stability. Our paper reports a case of a sporadic early-onset AD (sEOAD) patient of a 54-year-old Korean man, where a novel *APOE* Leu159Pro heterozygous mutation was revealed upon Whole Exome Sequence analysis. The proband's CSF showed downregulated levels of Aβ42, with unchanged Tau levels. The mutation is in the Low-Density Lipoprotein Receptor (LDLR) region of the APOE gene, which mediates the clearance of APOE lipoproteins. LDLR works as a high-affinity point for APOE. Studies suggest that APOE-LDLR interplay could have varying effects. The LDLR receptor pathway has been previously suggested as a therapeutic target to treat tauopathy. However, the APOE-LDLR interaction has also shown a significant correlation with memory retention. Leu159Pro could be an interesting mutation that could be responsible for a less damaging pattern of AD by suppressing tau-association neurodegeneration while affecting the patient's memory retention and cognitive performance.

## Introduction

Alzheimer's disease (AD) is prominent dementia instigating factor that affects cognitive abilities resulting in abnormalities like discrete diminution in thinking, memory, attention, or judgment (1). It accounts for 50–75% of dementia worldwide, affecting 44 million people. With the growing population in Asia, aging became an issue, and South Korea is one of the leading countries to witness such an irreversible form of illness in its population (2). While EOAD comprises 1–6% of all AD cases, sporadic EOAD (sEOAD) accounts for only 2% of all AD cases (2). There is a gap in understanding how familial EOAD (fEOAD) differs from sEOAD, however, studies show there is a strong genetic predisposition in fEOAD, which lacks in sEOAD cases despite showing a gradual memory decline that starts at a very early age. fEOAD results from mutations in one of the three genes: APP, PSEN1, and PSEN2. However, sEOAD does not have a deterministic risk factor that explains the development of AD at an early age. Four major genetic loci have been related to AD, *APOE* (Apolipoprotein E) on chromosome 19, *APP* (Amyloid precursor protein) on chromosome 21 (3), *PSEN1* (Presenilin-1), and *PSEN2* (presenilin-2) on chromosome 14 and 1, respectively (4–8). Among these mutations, *APP, PSEN1*, and *PSEN2* mutations represent only a minority of all patient's early-onset AD (EOAD) cases with a familial inheritance, suggesting that *APOE* (apolipoprotein E) on chromosome 19, may play an important role in disease onset in sporadic cases of EOAD (9, 10). EOAD has a clinical manifestation before 65 years of age. Although there is an overlap of phenotypes between LOAD and EOAD, EOAD shows more symptoms of language and visuospatial impairment, and executive dysfunction (11). APOE has two polymorph sites: Cys112 and Arg158. Their mutations could result in three main alleles of APOE: E2 (Cys112, Cys112), E3 (Cys112, Arg158), and E4 (Arg158, Arg158), and their heterozygous combinations (12, 13). The level of Aß42 in the cerebrospinal fluid determines the onset of preclinical AD and is associated with a strong predictive marker of cognitive decline in normal subjects (14–17), whereas total-tau and phosphorylated-tau are late-onset markers of AD (18). Low levels of Aβ42 indicate the early onset of AD even in cognitively normal people, making it the earliest marker for preclinical prediction of sEOAD (19–21).

## Case Presentation

**Figure 1:**
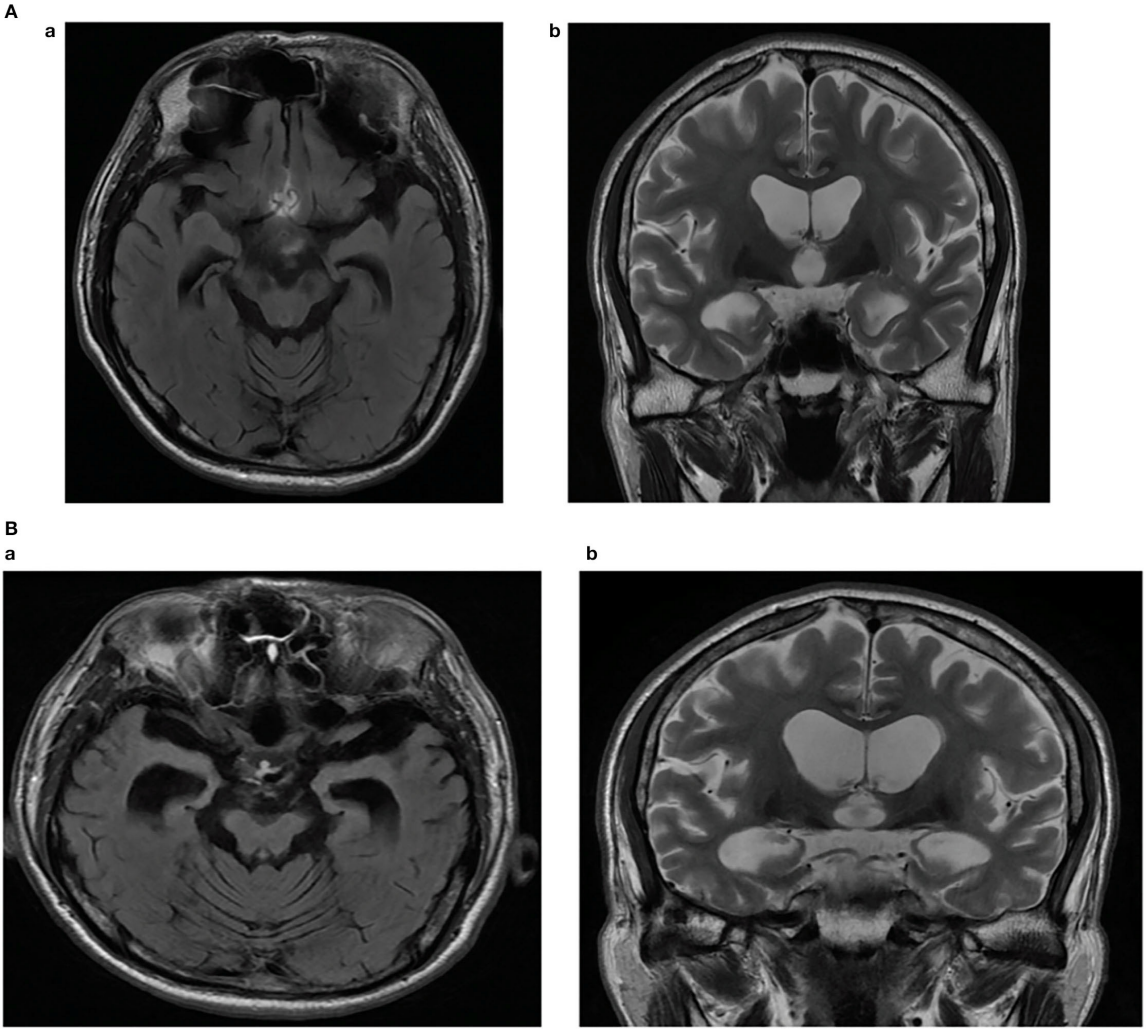
**(A)** Brain magnetic resonance imaging of the patient showing diffuse atrophy of the brain, more predominant medial temporal lobes. Fluid-attenuated inversion recovery (FLAIR) axial image (a), T2-weighted coronal image (b). **(B)** Brain magnetic resonance imaging (MRI) of the patient showing interval progression of diffuse (mainly frontotemporal) brain atrophy and marked medial temporal lobar atrophy after 18 months. Marked increased in size of with signal void in cerebral aqueduct, decreased in size of both basal ganglia compared to the MRI of the first visit are revealed. Fluid-attenuated inversion recovery (FLAIR) axial image (a), T2-weighted coronal image (b).

The proband patient was referred to the memory clinic of Konkuk University Medical Center at the age of 54, with a 2–3-month history of memory loss, executive dysfunction, and language dysfunction. He experienced difficulties in writing, planning tasks, and discussing a new topic. He started to forget topics he had discussed the previous day. Neuropsychological tests were conducted, and he scored 26 on the Mini-mental status examination (MMSE), 5 on the Clinical dementia rating scale (CDR), 4 on the Clinical Dementia Rating-Sum of Box score (CDR-SB), and 3 on the Global Deterioration Scale (GDS). Memory impairment with frontal/executive dysfunction and mild language dysfunction were revealed. Analysis of his APOE polymorphism revealed APOE 3/3. Brain magnetic resonance imaging (MRI) demonstrated diffuse atrophy of the brain, with more predominant medial temporal lobes, which is consistent with AD (Figure 1A). Because of his young age and clinical presentation with findings, EOAD was the most likely clinical diagnosis. About 5 mg of Donepezil was recommended with a gradual increase to 10 mg. Follow-up neuropsychological test revealed additional decreased attention and mild visuospatial dysfunction, and memantine 10 mg bid was added to his prescription. After 7 months of diagnosis, his activities of daily living started to further depreciate compared to the first visit. He often missed his usual subway stop at the university, which interfered with his daily duty of teaching or discussion. His long-term spatial memory started to impair to the point that he had difficulty remembering the nursing home, where his mother was living, and had to be accompanied by friends or other family members. Changes in behavior and personality started to get more pronounced. He would often scream and use profanity at his sister. He became more stubborn and would not let anybody interfere with his financial management and would often lose a check, or credit cards were suspended due to insufficient bank balance. He showed agitation and poor hygiene. Global cognitive assessments revealed declined daily activities of living; MMSE 25, CDR 1, CDR-SB 5.5, and GDS 4. After 18 months since the first diagnosis, the memory impairment of the patient worsened. The language function was further degraded, and only very short answers with mild dysarthria were possible. The MMSE, CDR, CDRS-SB, and GDS scores were 23, 1, 9, and 5, respectively. Follow-up MRI revealed interval progression of diffuse (mainly frontotemporal) brain atrophy and marked medial temporal lobar atrophy. A marked increase in the size of signal void in the cerebral aqueduct and a decrease in the size of basal ganglia were also compared to the MRI of the first visit, which supported the progression of the disease (Figure 1B).

Our patient scored 12(144) on the K-NPI (Korean version of Neuropsychiatric Inventory) test, while it decreased to 9(144) mid-year, and then saw a sharp increase to 44(144) in 2014 when he was diagnosed with EOAD. The detailed test scores are given in the table below (Table 1).

**Table 1:** Neuropsychiatric scores of the patient were marked as eating and sleeping changes, aberrant motor behavior, aggression, apathy, and disinhibition were identified over the course of his diagnosis.

**K-NPI Scores**	**2013-01-05**	**2013-07-30**	**2014-06-03**
Eating change	4	4	8
Sleeping change	–	–	8
Apathy	8	4	8
Aggression	–	1	–
Disinhibition	–	–	8
Aberrant motor behavior	–	–	12
Total K-NPI	12	9	44

*His total K-NPI scores were 44 with an increase in apathy, eating and sleeping changes, disinhibition, and aberrant motor behaviors*.

**Figure 2:**
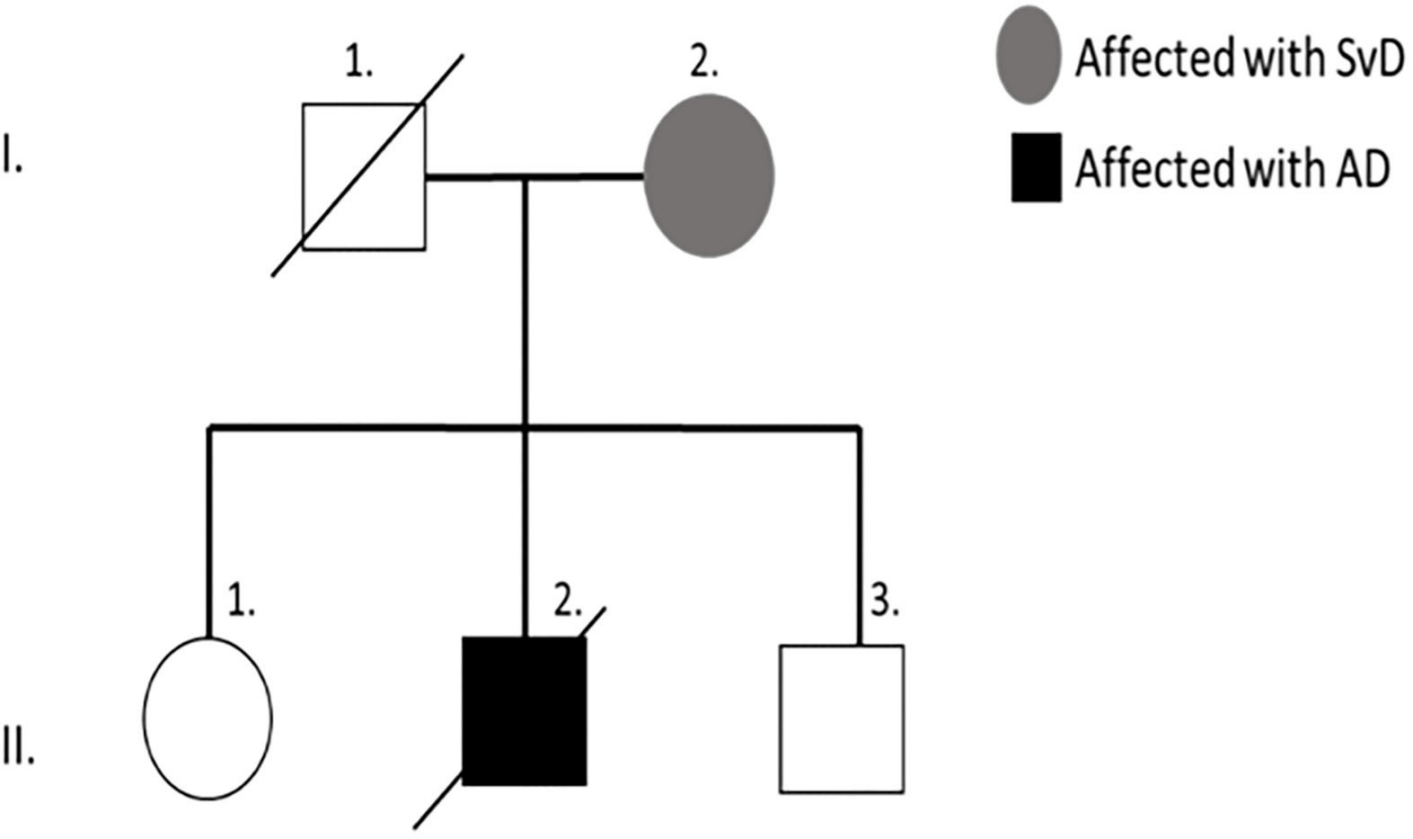
Family tree of proband patient (II-2).

The proband's mother (I-2) had been diagnosed with subcortical ischemic vascular dementia (SIVD) and the father (I-1) was deceased. The patient survived with an older sister (II-1) and a younger brother (II-3), both are currently unaffected (Figure 2).

After 19 months of initial diagnosis of dementia, the patient, unfortunately, suffered a heatstroke followed by subsequent pneumonia and died after 10 months following complications of bacterial meningitis and respiratory difficulty.

## Materials and Methods

This study was performed with the approval of the Institutional Review Board of Konkuk University School of Medicine and Konkuk University Medical Center (IRB no. KUH2021-03-016). Written informed consent was collected from the patient under study to allow clinical and genetic data to be utilized for medical research purposes. A probable diagnosis of early-onset Alzheimer's disease (EOAD) was actualized by the clinicians. The white blood cell and CSF samples of the patient were received in our laboratory. The concentrations of CSF Aβ42, T-tau, and P-tau181 were quantified using commercial ELISA kits (INNOTEST β-AMYLOID (1–42), INNOTEST hTAU- Ag, and INNOTEST PHOSPHO-TAU (181P), Fujirebio Europe, Gent, Belgium) according to the manufacturer's instructions. The patient's blood sample was purified from the Qiagen blood kit. Whole exome sequencing (WES) was performed on the patient by Novogene Inc (https://en.novogene.com; Beijing, China). DNA of the patient was extracted from the whole blood sample using standard laboratory protocols. DNA purification was checked using the NanoPhotometer spectrophotometer. Whole exome sequencing (WES) was performed on the Novaseq6000 platform with a pair-end 150 bp read length. The average coverage was 100X. The intricate genetic assessment was performed by NGS. The targeted gene panel test was reviewed for several causal and risk genes involved in different neurodegenerative diseases like Alzheimer's Disease, Parkinson's Disease, frontotemporal dementia, and prion disease (Supplementary Table 1) Standard Sanger sequencing was performed to validate the NGS records by Bioneer Inc., (http://eng.bioneer.com/home.aspx, Bioneer Inc. Daejeon, Korea). Data alignment was done using National Center for Biotechnology Information (NCBI) Blast (http://blast.ncbi.nlm.nih.gov/Blast.cgi) The variant was checked in the NCBI database genome browser for humans (https://www.ncbi.nlm.nih.gov/genome/gdv/) for its uniqueness. It was also monitored in 1,000 Genomes (https://www.internationalgenome.org/), GnomAD browser (https://gnomad.broadinstitute.org/), and in the Korean reference genome database (KRGDB) amongst 1,722 healthy Korean individuals. Mutations were screened by PolyPhen-2 (http://genetics.bwh.harvard.edu/pph2/), Sorting Intolerant from Tolerant (SIFT; http://sift.jcvi.org/), and PROVEAN (http://provean.jcvi.org/index.php) software, which performs predictions on the potential pathogenic nature of variants. Analysis by the ExPASy server (https://www.expasy.org/) was also carried out on three parameters: bulkiness, polarity, and hydrophobicity (Kyte and Doolittle scale). Crystal structure of APOE was downloaded from the Protein Data Bank (PDB; http://www.rcsb.org/pdb/) and mutation was generated by Discovery Studio 3.5 Visualizer software, designed by Accelrys. Normal (E3) and mutant (Leu159Pro) APOE were compared based on structural changes and intramolecular interactions.

## Results

Cerebrospinal fluid (CSF) analysis on the patient revealed very low concentrations of amyloid-beta 42, suggesting substantial progression of amyloid pathology (Table 2). However, his total tau and phosphorylated tau were not increased (Table 2). This could suggest possible protection against tauopathy.

**Table 2:** Cerebrospinal fluid (CSF) analysis on the patient compared to 6 cognitive normal samples revealed downregulated Aβ42; Total-tau and Phosphorylated-tau showed decreased values, suggesting tauopathy did not start.

**Sample**	**Aβ42 (pg/mL)**	***t*** **-tau (pg/mL)**	**p-tau (pg/mL)**
Normal1	1,404.4	126.1	34.5
Normal2	1,392.9	95.8	29.8
Normal3	891.5	255.8	48.1
Normal4	1,056.2	129.4	40.0
Normal5	1,387.3	126.3	37.9
Normal6	1,702.4	309.4	58.4
Patient	285.8	356.3	25.4

*Aβ42, amyloid-β 42; t-tau, Total tau; p-tau, phosphorylated tau*.

**Figure 3:**
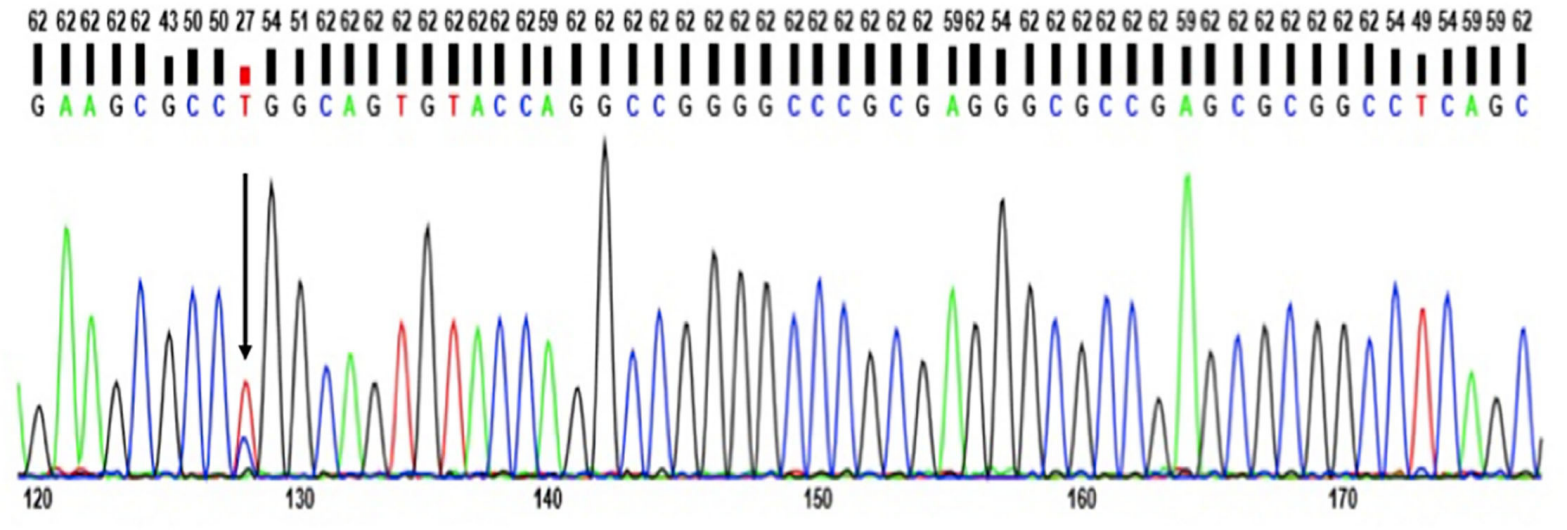
Standard sequencing of APOE Leu159Pro mutation showing it is a heterozygous mutation.

Whole Exome Sequencing (WES) revealed no pathogenic mutations in APP, PSEN1, or PSEN2 genes. No rare variants were found in AD risk genes either, such as SORL1, ABCA7, or CLU (Supplementary Table 2). In addition, no pathogenic variants appeared in other disease-associated genes including PD, FTD, and Prion disease. A novel heterozygous T>C exchange at chromosome 19 g.19:45412083, c.530T>C; Leu159Pro was recognized and validated in the APOE-coding region with WES, as well as Sanger Sequencing (Figure 3).

This is a Leucine to Proline substitution in APOE exon 4 at position 159, which was not found in either of the databases like KRGDB, 1,000 Genome, or ExAC. APOE Leu159Pro was suggested as deleterious or damaging by MutationTaster, PolyPhen2, and SIFT tools. SIFT and PolyPhen2 revealed this mutation as *pathogenic* with HumDiv and HumVar scores, whereas MutationTaster predicted this as a *disease-causing* mutation with a probability value of prediction security of 0.999. Multiple sequence alignment by PolyPhen2 suggested that Leucine159 is conservative among several primate species, including *Hylobates leucogenys, Macaca fascicularis, Papio Anubis*, or *Callithrix jacchus*. The majority of the mammalian species also had leucine in the same position: California sea lion (*Zalophus californianus*), Naked mole-rat (*Heterocephalus glaber*), and mouse (*Mus Musculus*). Proline was not found at the homologous position of APOE-like sequences of any species; however, Methionine and Isoleucine were observed in Rabbit (Oryctolagus cuniculus) and Duckbill platypus (Ornithorhynchus anatinus), respectively.

**Figure 4:**
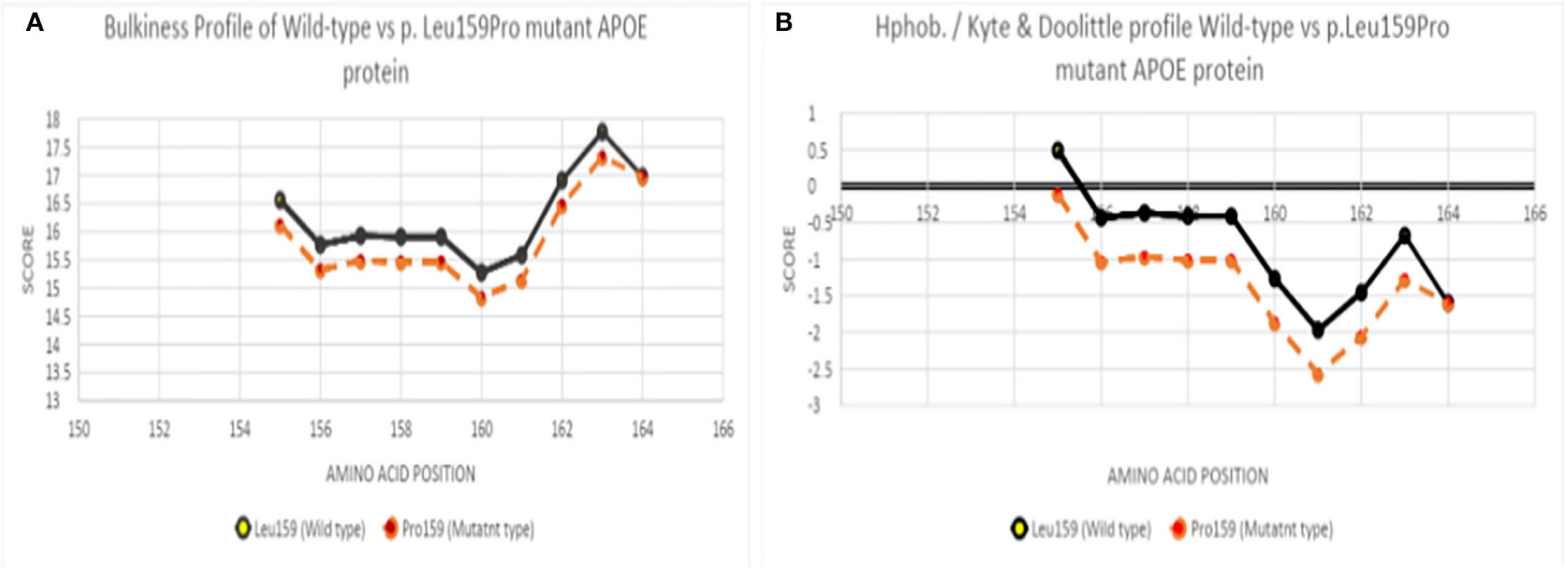
**(A)** Bulkiness profile of wild-type and Leu159Pro mutant APOE protein, **(B)** Hydrophobicity profile of wild-type and Leu159Pro APOE mutant protein.

**Figure 5:**
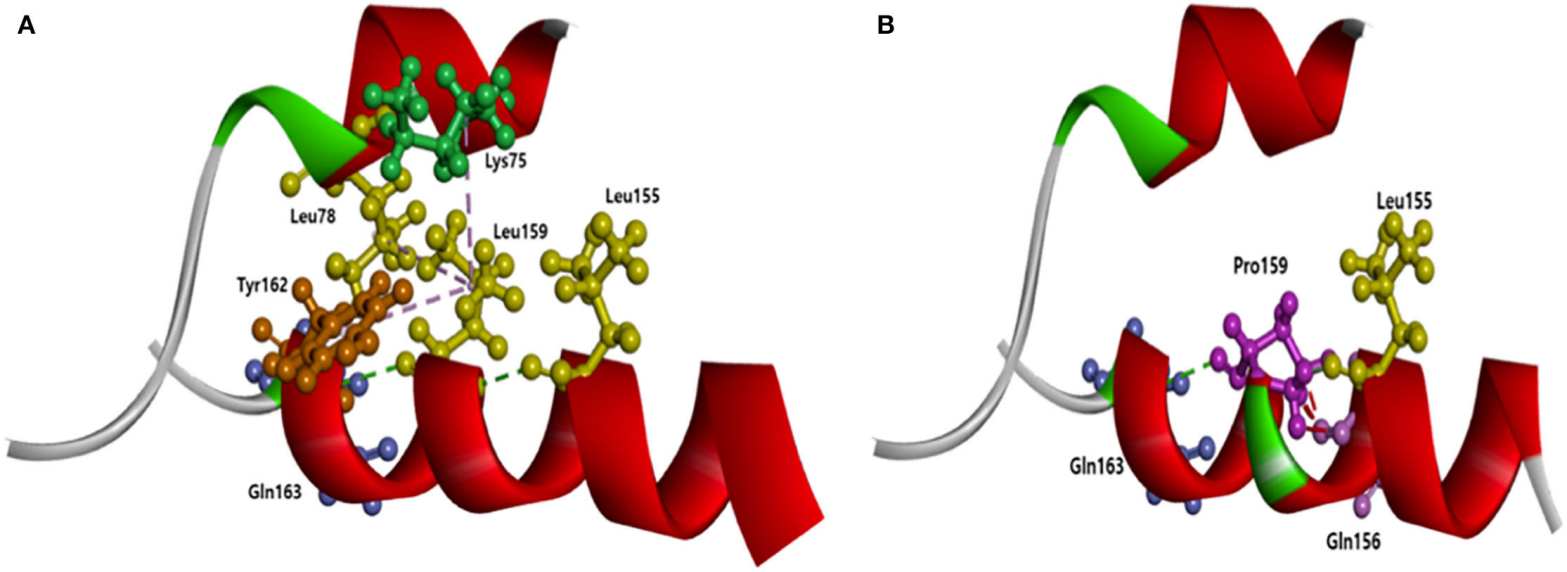
Intramolecular interactions of APOE with Leu159, compared to the APOE with Pro159. Pro159 may significantly disturb the intramolecular structure of APOE by the loss of contact between Helix-1 and Helix-4. **(A)** Normal APOE with Leu159; **(B)** Mutant APOE with Pro159.

The ExPasy tool showed that scores of bulkiness significantly dropped from 15.903 to 15.462 at Leu159Pro, and nearby amino acids were also affected as result (Figure 4A). Kyte and Doolittle Hydrophobicity scores revealed that the score of hydropathicity on Pro159 also dropped from −1.011 to −0.411 (Pro159), suggesting that this mutation is less hydrophobic (Figure 4B). Structure prediction of APOE3 Leu159Pro revealed that proline could disturb the structure of Helix-4 since it could result in a kink inside the alpha-helix (Figures 5A,B).

This kink may change the shape of the helix, resulting in an abnormal helical motion. Intramolecular interactions could be significantly changed due to APOE3 Leu159Pro. In normal APOE3, Leu 159 could form hydrogen bonds with two nearby residues: Leu155 and Gln163. Additionally, Leu159 could form hydrophobic interactions with Tyr162 and two residues in helix-1 (Leu78 and Lys75). Pro159 could destroy the contact between Helix-4 and Helix-1 since hydrophobic interactions with Leu18 and Lys75 would be lost. In addition, Pro159 could form strong hydrogen bonds with Gln156. The hydrogen bonds between Pro159, Leu155, and Gln163 remain, but the distance between Pro159 and these residues may alter.

The ClueGo pathway analysis was performed to predict putative interaction among neurodegenerative genes and their possible common pathways. Among the gene interactions, APOE was found to play a central role in three pathways (Supplementary Figure 1). First, along with ABCA7, SORL1, and PON1, APOE may play a role in the positive regulation of cholesterol efflux. Second, APOE could interact with several genes (including EPHA1, DBN1, GIGYF2, NOTCH3, FIG1, OPTN, PTK2B, SETX, and MAPT), and play a role in the negative regulation of fatty acid oxidation. Finally, along with ABCA7, LRRK2, HIP1R, SINJ1, and APLP2, APOE may play a role in the positive regulation of endocytosis.

## Discussion

APOE is a 299 amino acids long single-chain polypeptide glycoprotein, that binds to the receptors of the cell surface in the low-density lipoprotein (LDL) receptor family. It helps in transporting lipid and lipid metabolism (22). Human APOE has three main polymorphic variants APOE2, APOE3, and APOE4 depending on the Cys-Arg substitute residues at positions 112 and 158 (23). APOE, a two-domain structure protein, activates the LDL receptor (24). The N-terminal domain consists of residues 1–191 and the C-terminal domain consists of 192–299. These two domains relate to a hinge. The N-terminal binds to the LDL receptor while the C-terminal binds to hyper-triglyceridemic very low-density lipoprotein (HVLDL) (25). Site 1–191 contains the receptor-bindingding domain in the Helix4 at residues 130–161 (26). Patients with APOE E4 allele were found to have amyloid depositions an in early age (40s−50s), while also resulting in glucose hypermetabolism in preclinical age of 30s (27, 28). The Association of APOE and EOAD remained unclear, but its role in EOAD may not be ruled out, as mutant APOE along with other putative genetic variants may result in an early onset of the disease formation (29). For example, APOE E4 along with PSEN1 Glu280Ala resulted in earlier disease onset (30), however, APOE E2 with PSEN1 Glu280Ala delayed the age of onset of the disease (31).

**Figure 6:**
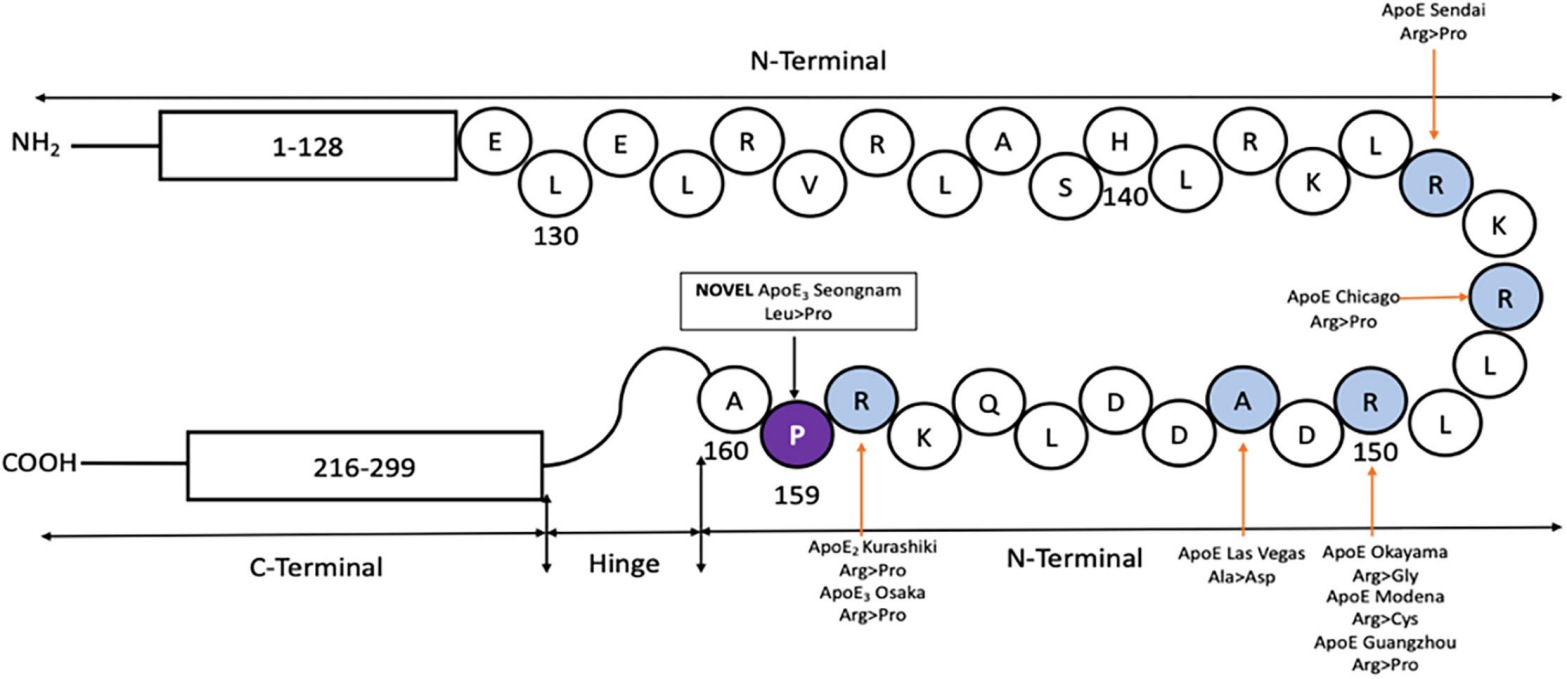
Protein map of APOE, showing the N and C terminal connected with a hinge. Previously reported mutations in the LDLR site (shaded in blue) are shown, along-with the novel APOE3 Leu159Pro (shaded in violet with the mutated codon) found in a Korean male patient with EOAD.

Oikawa et. al discovered APOE Sendai, a G>C point mutation at residue codon 145, with an Arginine to Proline substitution (32). Sam et al. reported APOE Chicago, a G>C point mutation at residue codon 147, with an Arginine to Proline substitution (33). Kinomura et al. reported APOE Okayama, a G>C substitution at codon 150, with an Arginine to Glycine replacement (22). Cautero et al. described a novel APOE Modena, a C>T homozygous mutation at residue codon 150, with an Arginine to Cysteine change (34). Luo et al. identified APOE Guangzhou, a G>C nucleotide substitution at residue codon 150, with an Arginine to Proline exchange (35). Bomback et al. presented a novel APOE Las Vegas mutation, a C>A point nucleotide change at codon 152, with an Alanine to Asparagine exchange (36). Tokura et al. reported a case of APOE2 Kurashiki novel mutation, a G>C substitution at codon 158 with Arginine to Proline change, while Mitani et al. reported a case of APOE3 Osaka novel mutation, also a G>C point mutation at codon 158 with Arginine to Proline substitution (37, 38). APOE plays a crucial role in mediating lipoprotein clearance by binding to cell surface receptors of LDLR (39). Until now, most of the mutations reported on the LDLR site of 135–160 (Figure 6) are associated with a rare disease called Lipoprotein Glomerulopathy (LPG) (40) which results from an increased APOE serum level (41). There are several reported cases with APOE but asymptomatic for LPG or renal failure (42–44). Leu159Pro lies next to APOE Osaka mutation in the lipid-binding region and alters the conformation of APOE. It might result in increased aggregation of Aβ as well as specific effects like cholesterol efflux on lipid metabolism.

Several common variants were found among the analyzed genes, included in our gene panel (Supplementary Tables 1, 2). Even though the majority of these variants were common, their impact on disease onset may not be ruled out. It may be possible that these variants in risk genes may interact and possibly act as risk modifiers (45, 46). ClueGo mapping was performed on mutation-carrying genes to predict their possible interactions. APOE may interact with several genes and was predicted to play a role in three pathways: (1) positive regulation of cholesterol reflux (2) negative regulation of fatty acid oxidation, and (3) positive regulation of endocytosis.

APOE may highly impact in positive regulation of cholesterol efflux along with ABCA7, SORL1, PON1, and APOC4. In ABCA7, three common missense mutations were found: Glu188Gly, Gly1527Ala, and Ala2045Ser. Among these variants, Gly1527Ala was found to reduce the ABCA7 expression and could play a role as a risk modifier (47, 48). Mutant ABCA7 and APOE were suggested to play a role in cognitive dysfunctions, amyloid generation, and senile plaque formation (49, 50). Both ABCA7 and APOE were confirmed to play a role in lipid homeostasis and cholesterol metabolism. Lower ABCA7 expression near blood-brain barrier (BBB) may reduce the APOE expression. Lower ABCA7 and APOE expression may result in decreased cholesterol exchange across blood-brain barrier (51, 52). Two common variants were found in SORL, Gln1074Glu, and Val1967Ile, both were suggested to be common benign variants (53). SORL1 could play a role as an APOE receptor and induce the cholesterol intake into neurons (54, 55). PON1 was also found to interact with APOE through the cholesterol pathway. APOE was suggested to enhance PON1 activity and stability. Their interaction may result in anti-atherogenic effects, such as inhibiting LDL cholesterol oxidation or enhancing the macrophage cholesterol efflux (56). Further investigations are needed on how APOE and APOC4 could interact to impact cholesterol metabolism (57). However, abnormal expression of APOC genes may be involved in human hyperlipidemias (58).

The APOE also interacts with several proteins to play a role in fatty acid oxidation. APOE dysfunctions may impair fatty acid transport, and reduce the fatty acid oxidation, resulting in fatty acid aggregation in astrocytes and brain (59). The third function APOE could be involved in the regulation of endocytosis (60). The role of common variants in the case of APOE Leu159Pro mutation may not be ruled out as these variants could interact as risk modifiers and impact the disease-related pathways.

It was previously reported that the APOE4 allele increases susceptibility to AD, as well as to an earlier onset of the disease (61). However, the consideration that APOE3 is neutral compared to APOE4, is not based on their contributions to pathogenesis. Holtzman et al. demonstrate how any allele of APOE can interpose pronounced tauopathy (62). AD is a slow developing disease starts with the accumulation of amyloid plaques in the brain which reduces Abeta42 in cerebrospinal fluid (CSF) (63). Tauopathy occurs over several years. Mutations in the LDLR domain of the APOE gene may disrupt the protein's ability to traffic lipids.

In this report, we described a Korean patient with sporadic EOAD who had long-term spatial memory impairment along with executive dysfunction, and language impairments. Mutations in other AD-related genes were absent, but there's a novel mutation Leu159Pro in the LDLR domain of APOE. His CSF analysis revealed a pronounced downregulation of Aβ42 levels, however his t-tau, and p-tau levels were not upregulated. This would infer that the patient had significant amyloid pathogenesis which contributed to his dementia, and long-term spatial memory impairment, however, tauopathy did not start. This could be possible due to the Leu159Pro mutation in the LDLR domain of APOE. The LDLR receptor interacts with all the different isoforms of APOE in the brain with different binding attractions (64, 65). The interplay of APOE and LDLR affects spatial memory, independent of APOE isoforms, associated with AD (66). While APOE has been studied profoundly about its disease-modifying mechanism in AD, the effect of APOE mutations in the LDLR domain in the brain has been understudied.

We hypothesize that Leu159Pro promotes lipoprotein metabolism and APOE turnover, by increasing LDLR expression. This results in an intracellular blockage resulting in aberrant accrual of Aβ plaques in the brain. This overexpression of Aβ plaques should lead to tau-related neurodegeneration. However, LDLR has a protective effect on tauopathy (62). Patients with dementia often display metabolic dysfunction with altered liver proteins. However, little is known about their cumulative effect on cognition (10). Our patient showed fewer signs of memory abilities despite exceedingly high levels of amyloid plaques, and low levels of tau build-up, similar to Christchurch homozygote mutation (67), however, it is interesting to note that similar to Christchurch heterozygous mutation, our patient too exhibited signs of spatial disorientation and progressive cognitive decline with an early onset of the disease (68). Leu159Pro may downgrade the ability of APOE to bind to specific factors essential to yielding tau tangles. Clinical information such as postmortem examination, tau-pet imaging, and detailed lipid profile since the onset of the disease condition are not currently available, which limits the understanding of the contribution of this new variant in lipid homeostasis as well as the formation of tau fibrils.

In conclusion, mutations in the LDLR domain of APOE could be a protective target to tau-associated neurodegeneration, while increasing the risk of amyloid load in sEOAD. Additional studies are required to better understand this hypothesis to produce a more definite answer to whether mutations in the LDLR domain of APOE could indeed be an interesting area to study about sporadic EOAD.

## Data Availability Statement

The datasets presented in this study can be found in online repositories. The names of the repository/repositories and accession number(s) can be found below: FigShare: https://doi.org/10.6084/m9.figshare.16627975.v2 (Bagaria, Jaya (2021): APOE Leu159Pro Sanger Sequencing.figshare.

## Ethics Statement

The studies involving human participants were reviewed and approved by Institutional Review Board of Konkuk University School of Medicine and Konkuk University Medical Center (IRB no. KUH2021-03-016). The patients/participants provided their written informed consent to participate in this study. Written informed consent was obtained from the individual(s) for the publication of any potentially identifiable images or data included in this article.

## Author Contributions

JB, YM, and SA: conceptualization. YM: data curation. JB: formal analysis, methodology, and software. SA: funding acquisition and resources. JB, YM, and EB: investigation. KS: CSF analysis. YM, SA, and SH: project administration. EB, SA, SK, and SH: supervision and writing—review and editing. YM and EB: validation. EB: visualization. JB and YM: writing—original draft. All authors contributed to the article and approved the submitted version.

## Funding

This research was funded by Basic Science Research Program through the National Research Foundation of Korea (NRF) funded by the Ministry of Education (2021R1A6A1A03038996), Brain Research Program of the National Research Foundation (NRF) funded by the Ministry of Science & ICT (NRF-2018M3C7A1056571), and Gachon University research fund of 2021 (GCU-202008480012).

## Conflict of Interest

The authors declare that the research was conducted in the absence of any commercial or financial relationships that could be construed as a potential conflict of interest.

## Publisher's Note

All claims expressed in this article are solely those of the authors and do not necessarily represent those of their affiliated organizations, or those of the publisher, the editors and the reviewers. Any product that may be evaluated in this article, or claim that may be made by its manufacturer, is not guaranteed or endorsed by the publisher.
