# Supplementary material for: Whole Exome Sequencing Reveals a Novel APOE Mutation in a Patient With Sporadic Early-Onset Alzheimer's Disease
Source: Front Neurol. 2022 Jun 10;13:899644. doi: 10.3389/fneur.2022.899644 (PMC9226417; doi:10.3389/fneur.2022.899644)
Supplement: Supplementary file 1 [file Table_1.DOCX]

**SUPPLEMENTARY FILES**

**Supplementary table 1.** Gene panel used in the analysis.

| **Genes, used in the gene panel** |
| --- |
| A2M, ABCA7, ABI3, ACTB,  ACE, ADAM10, AIF1, AKT1, ALB,  ALS2, ANG, APBB1, APCS, APH1A, APLP2, APOC2, APOC4,APOE, APP, ATP13A2, ATXN1, ATXN2, BACE1, BIN1,  BST1. C9ORF72, CASP3, CASS4, CD2AP, CCNF, CD33, CDH12, CDH18,  CDK5, CHAT,  CHMP2B, CLU, CR1,  CREB1,  CSF1R , CST3, CTSB, CYP7B1,  CTNNA3, CTSA, CTSD, DAO, DBN1, DCTN1, DLG4,  DNMBP, DSG2,  EPHA1,  EWSR1, FBXO7, FERMT2,  FIG4,  FGF20, FUS,  GAB2,  GAK, GBA,GAPDH, GFAP, GIG25,  GIGYF2,  GPNMB, GRN, GPX4, GRIN2B, GRIN3B, GSK3A, GSK3B,  HIP1R, HLA-DRB1, HLA-DRB5,  HNRNPA1, HNRNPA2B1, HSPG2, HTRA1,  HTT, IAPP, IDE, IL6, INS,  LAMP3, LPR6,  LRRK2, MAOB, MAPT, MARK4, MS4A4A,  MEF2C, MS4A6E, MTND1, MTHFD1L,  MTND2, NCSTN, NEFL, NEK1,   NGF, NME8,  NOTCH3, OPTN,  PARK2,  PARK7,  PFN1, PHF1, PICALM, PILRA, PILRB, PINK1,  PLA2G6 PLD3, PON1, PPP5C, PPT1,  PRNP, PSEN1, PSEN2, PSENEN, PTK2B,  PVRL2, RALGOS2, RBFOX3, RELB, RIN3, S100A9,  SETX , SIRT1,  SIGMAR1, SLC24A4, SNCA, SOD1, SORL1, SPAST,  SPG11 SQSTM1,  STBD1, STK39, STX1B, SYT11, SYNJ1, SYP,  TAF15, TARDBP,  TBK1,  TM2D3,  TMEM106B.TNF, TOMM40, TREM2, TREML2, TTR, UBQLN2,  UNC5C,  VAPB, VCP,VPS35,  YWHAQ, ZCWPW1 |

**Supplementary table 2.** Mutations, found in the patient by neuro-degenerative genetic analysis

| **GeneName** | **AAChange** | **RS ID** | **Alternate Allele Frequency** | | | | **Pathogenicity Predictions** | |
| --- | --- | --- | --- | --- | --- | --- | --- | --- |
|  |  |  | **1000g** | **ExAC_ALL** | **EAS** | **Korean** | **SIFT** | **Polyphen2** |
| A2M | N639D | rs226405 | 0.997804 | 0.9995 | 1 | 1 | 0.758,T | 0.0,B |
| ABCA7 | E188G | rs3764645 | 0.3996 | 0.4838 | 0.4932 | 0.4352 | 0.647,T | 0.358,B |
|  | G1527A | rs3752246 | 0.8255 | 0.8405 | 0.655 | 0.6654 | 0.877,T | 0.0,B |
|  | A2045S | rs4147934 | 0.605 | 0.7317 | 0.438 | 0.4535 | 0.962,T | 0.057,B |
| ABI3 | F209S | rs616338 | 0.998403 | 0.9943 | 1 | 1 | 1.0,T | 0.0,B |
| AKT1 | I75M | rs587778019 | NA | 1.66E-05 | 0.0017 | 0 | 0.044,D | 0.999,D |
| ALS2 | V368M | rs3219156 | 0.8966 | 0.9106 | 1 | 0.9988 | 0.191,T | 0.006,B |
| APLP2 | D632N | rs3740881 | 0.06909 | 0.0211 | 0.0659 | 0.0475 | 0.153,T | 0.124,B |
| APOC4 | L36P | rs1132899 | 0.664337 | 0.5921 | 0.6727 | 0.6923 | NA | 0.0,B |
|  | L96R | rs5167 | 0.439297 | 0.3892 | 0.5388 | 0.5205 | NA | 0.0,B |
| APOE | L159P | NA | NA | NA | NA | NA | 0,D | 1,D |
| ATF6 | M67V | rs1058405 | G=0.1669 | G=0.235 | G=0.2955 | G=0.3406 | 0.1676,T | 0.372,B |
|  | A145P | rs2070150 | C=0.1591 | C=0.1302 | C=0.2955 | C=0.3352 | 0.435,T | 0.364,B |
|  | P157S | rs1135983 | T=0.1869 | T=0.1378 | T=0.3427 | T=0.3689 | 0.647,T | 0.206,B |
| ATP13A2 | A1072T | rs3170740 | 0.3313 | 0.5065 | 0.279 | 0.3413 | 1.0,T | 0.001,B |
| ATXN1 | Q213H | rs3817753 | NA | 0.0031 | 0 | 0.2704 | 0.072,T | 0.001,B |
| ATXN2 | S248N | rs7969300 | 0.1795 | 0.0905 | 0.5680 | 0.5474 | 0.136,T | 0.0,B |
| BACE1 | C412R | rs539765 | 0.000551 | 0.000334 | 0 | 0 | 1.0,T | 0.0,B |
| BST1 | G36A | rs2302468 | 0.0761 | 0.0646 | 0.04 | 0.2165 | 0.034,D | 0.999,D |
|  | R145Q | rs2302464 | 0.0791 | 0.0485 | 0.2152 | 0.2437 | 0.118,T | 1.0,D |
| CHAT | D47E | rs3810948 | 0.108826 | 0.0386 | 0.429 | 0.3580 | 0.0,D | 0.122,B |
|  | D7N | rs1880676 | 0.161542 | 0.2112 | 0.1463 | 0.1577 | 0.0,D | NA |
|  | A2T | rs3810950 | 0.160543 | 0.2269 | 0.1461 | 0.1386 | 0.588,T | 0.13,B |
|  | V343M | rs4838544 | 0.966454 | 0.9896 | 1 | 1 | 1.0,T | 0.048,B |
| CALML6 | W60R | rs28581776 | C=0.2147 | C=0.14051 | C=0.191 | C=0.1533 | 0.116, T | 0.2,B |
| CAPN2 | D22E | rs25655 | 0.988 | 0.996 | 1 | 1 | 0.996,T | 0.999,B |
|  | R205C | rs149096348 | 0.00119 | 0.0003 | 0.0060 | 0.0033 | 0.0059,D | 1.0,D |
| CASP9 | Q221R | rs1052576 | T=0.4151 | T=0.4630 | 0.6176 | 0.612 | 0.392,T | 0.651,B |
|  | A28V | rs1052571 | 0.5847 | 0.503 | 0.6253 | 0.5792 | 0.5026,T | 0.6836,B |
| CR1 | H1658R | rs2274567 | 0.2943 | 0.251 | 0.277 | 0.1932 | 0.897,T | 0.995,D |
|  | T1858M | rs3737002 | 0.2488 | 0.275 | 0.4236 | 0.4273 | 0.021,D | 1.0,D |
|  | T2060S | rs4844609 | 0.995 | 0.9853 | 1 | 1 | 1.0,T | 0.003,B |
|  | I2065V | rs6691117 | 0.4934 | 0.3341 | 0.2298 | 0.2102 | 1.0,T | 0.001,B |
|  | T2419A | rs2296160 | 0.8281 | 0.8159 | 0.56 | 0.6522 | 1.0,T | 0.0,B |
| DBN1 | S555P | rs28538572 | 0.998802 | 0.9995 | 1 | 1 | 1.0,T | 0.0,B |
|  | P519L | rs180961453 | 0.000799 | 0.0003 | 0.0005 | 0.0086 | 0.097,T | 0.618,P |
| DSG2 | R773K | rs2278792 | 0.24 | 0.2676 | 0.4874 | 0.4853 | 0.383,T | 0.026,B |
|  | P927L | rs146402368 | 0.0004 | 0.0003 | 0 | 0.0137 | 0.001,D | 0.972,D |
| EPHA1 | M900V | rs6967117 | 0.9603 | 0.9366 | 0.9988 | 1 | 1.0,T | 0.0,B |
|  | V849M | rs772410524 | NA | 8.69E-06 | 0 | NA | 1.0,T | 0.449,B |
|  | V160A | rs4725617 | 0.9435 | 0.9284 | 0.9893 | 0.9902 | 0.246,T | 0.0,B |
| FBXO7 | M115I | rs11107 | 0.4874 | 0.44 | 0.6977 | 0.7533 | 1.0,T | 0.0,B |
| FIG4 | V654A | rs9885672 | 0.4479 | 0.2753 | 0.3712 | 0.3672 | 0.833,T | 0.0,B |
| GALP | I72M | rs3745833 | 0.3255 | 0.2174 | 0.2330 | 0.2249 | 0.009,D | 0.905,P |
| GRIN3A | D835N | rs10989563 | 0.131589 | 0.2034 | 0.0457 | 0.0445 | 0.11,T | 0.999,D |
|  | G487R | rs10989589 | 0.265375 | 0.3597 | 0.1953 | 0.1940 | 0.089,T | 0.956,P |
|  | V362M | rs10989591 | 0.319688 | 0.3033 | 0.1115 | 0.1465 | 0.025,D | 0.429,B |
| HIP1R | V782M | rs2271051 | 0.1585 | 0.0988 | 0.211 | 0.2536 | 0.53,T | 0.0,B |
| HSPG2 | A1503V | rs897471 | 0.691494 | 0.7463 | 0.888 | 0.8865 | 0.056,T | 1.0,D |
|  | N765S | rs989994 | 0.929912 | 0.9811 | 1 | 1 | 0.246,T | 0.003,B |
|  | M638V | rs1874792 | 0.973642 | 0.9931 | 1 | 1 | 1.0,T | 0.0,B |
| HTRA1 | A20V | rs369149111 | 0.0507 | 0.0321 | 0.24 | 0.1382 | 0.633,T | 0.001,B |
| LRP6 | V1062I | rs2302685 | 0.8856 | 0.8474 | 0.9304 | 0.9273 | 1.0,T | 0.0,B |
| LRRK2 | R50H | rs2256408 | 0.9692 | 0.9911 | 1 | 1 | 1.0,T | 0.0,B |
| MAPT | Y441H | rs2258689 | 0.3129 | 0.2752 | 0.6211 | 0.6283 | 0.978,T | 0.001,B |
| MS4A6E | I6V | rs2304935 | 0.2582 | 0.3185 | 0.2851 | 0.3027 | 1.0,T | 0.0,B |
|  | T10A | rs2304934 | 0.2582 | 0.3184 | 0.281 | 0.3031 | 0.086,T | 0.106,B |
| NEK1 | E752G | rs34099167 | 0.105 | 0.1403 | 0.1673 | 0.1621 | 0.088,T | 0.971,D |
| NME8 | C208R | rs10250905 | 0.7434 | 0.7383 | 0.541 | 0.5495 | 0.046,D | 0.001,B |
| NOTCH3 | A2223V | rs1044009 | 0.6294 | 0.7591 | 0.583 | 0.5844 | 0.175,T | 0.001,B |
| OPTN | M98K | rs11258194 | 0.0787 | 0.0452 | 0.1062 | 0.0842 | 0.925,T | 0.001,B |
|  | K322E | rs523747 | 0.0066 | 0.0027 | 0 | 0 | 1.0,T | 0.0,B |
| PHF1 | R304K | rs3116713 | 0.977636 | 0.9515 | 0.999 | 0.9997 | 0.553,T | 0.002,B |
| PON1 | Q192R | rs662 | 0.4571 | 0.377 | 0.6408 | 0.6618 | 0.779,T | 0.0,B |
| PTK2B | K838T | rs751019 | 0.3646 | 0.437 | 0.3384 | 0.3512 | 0.439,T | 0.014,B |
| SETX | S2612G | rs3739927 | 0.1635 | 0.0852 | 0.4040 | 0.4365 | 0.751,T | 0.0,B |
|  | I2587V | rs1056899 | 0.5387 | 0.3926 | 0.7494 | 0.7662 | 1.0,T | 0.0,B |
|  | T1855A | rs2296871 | 0.4431 | 0.2758 | 0.7169 | 0.741 | 0.984,T | 0.0,B |
| SORL1 | Q1074E | rs1699107 | 0.9848 | 0.9949 | 1 | 1 | 1.0,T | 0.0,B |
|  | V1967I | rs1792120 | 0.9794 | 0.9953 | 1 | 1 | 1.0,T | 0.0,B |
| SYT11 | Q48H | rs822522 | 0.9547 | 0.988 | 1 | 1 | 0.866,T | 0.0,B |
| TM2D3 | TL6R | rs2939587 | 0.9934 | 0.9798 | 1 | 1 | 0.466,T | 0.0,B |
| TMEM106B | T185S | rs3173615 | 0.595 | 0.4916 | 0.6389 | 0.6577 | 0.214,T | 0.043,B |
| TREML2 | S144G | rs3747742 | 0.313698 | 0.3261 | 0.3582 | 0.3420 | 0.218,T | 0.002,B |
| UNC5C | A841T | rs34585936 | 0.018371 | 0.0186 | 0.0282 | 0.0370 | 0.002,D | 0.996,D |
| **INDELS** | **AAChange** | **RS ID** | **Alternate Allele Frequency** | | | | **Pathogenicity Prediction** | |
|  |  |  | **1000g** | **ExAC_ALL** | **EAS** | **Korean** | **SIFT** | **Polyphen2** |
| APLP2 | p.221_222del | rs3837393 | 0.091454 | 0.0878 | NA | NA | NA | NA |
| ATXN2 | p.188_189del | rs10560189 | 0.951078 | 0.5912 | 0.86 | NA | NA | NA |
| CTSA | p.29_29del | rs10582052 | 0.649361 | 0.6432 | NA | NA | NA | NA |
| GIGYF2 | p.P1210fs | rs371622656 | NA | 0.0688 | NA | 0.2877 | NA | NA |
|  | p.P1210fs | rs775324034 | NA | 0.174 | NA | NA | NA | NA |
| GRIN3B | p.G466fs | rs10666583 | 0.159145 | 0.2448 | 0.082 | NA | NA | NA |
|  | p.1028_1036del | rs142516571 | 0.178714 | 0.2579 | 0.08 | 0.0715 | NA | NA |
| HIP1R | p.417_421del | rs544081892 | 0,000998 | 0,0005 | 0 | NA | NA | NA |
| HTT | p.18_19del | rs374076986 | 0.377796 | 0.1754 | 0 | NA | NA | NA |
| RIN3 | p.967_967del | rs570458246 | NA | 0.6674 | 0.60 | NA | NA | NA |
| SYNJ1 | L1406delinsNTL | rs57257560 | 0.571086 | 0.512 | 0.24 | 0.5513 | NA | NA |

Abbreviations: *T* tolerated; *P* probably damaging; *B* benign; *D* damaging


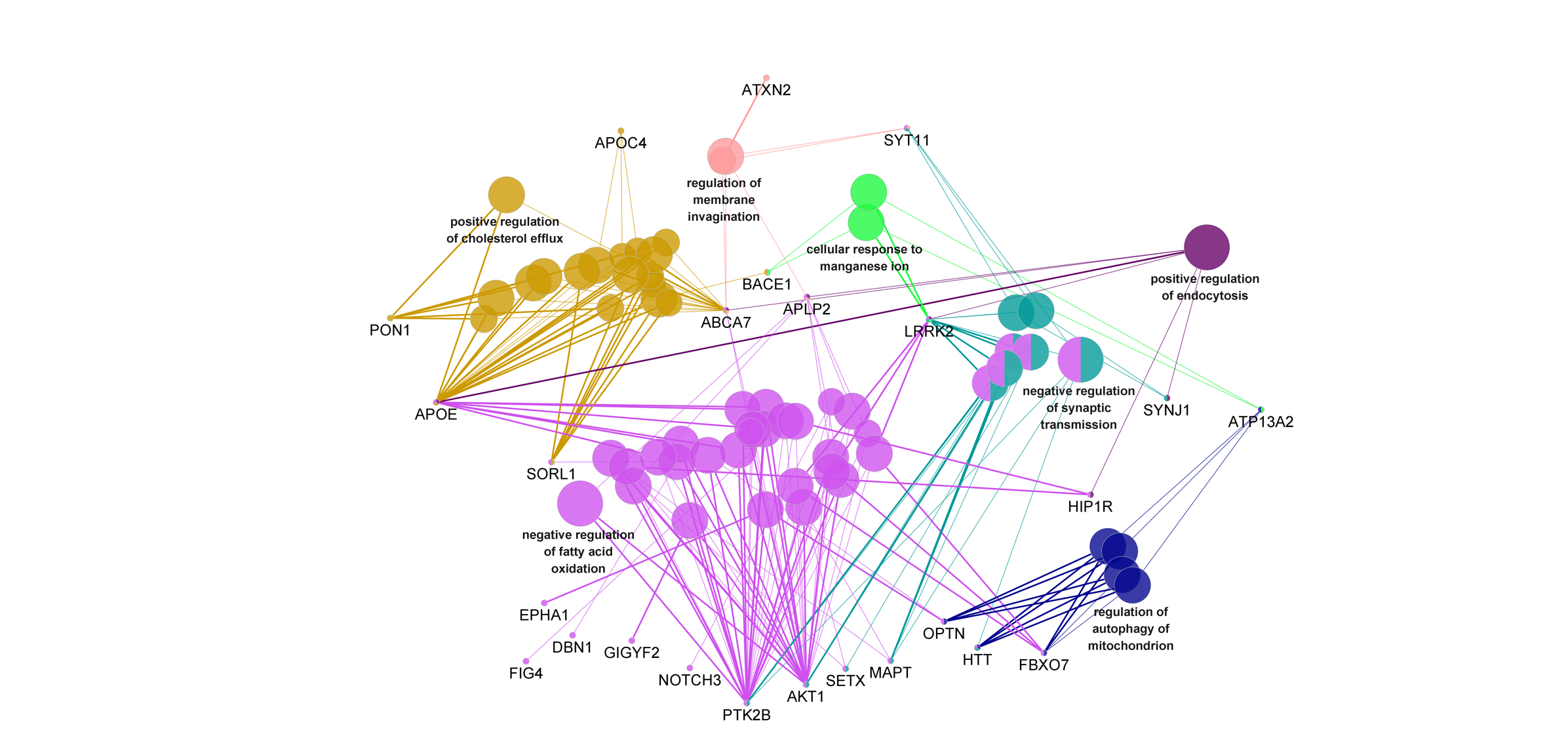


Supplementary Figure 1. ClueGO analysis of neurodegenerative genes showing their putative interaction and possible pathways they are involved in.
